# The Effectiveness of Attribution Retraining on Health Enhancement of Epileptic Children

**Published:** 2016

**Authors:** Tahereh NAJAFI FARD, Masoume POURMOHAMADREZATAJRISHI, Firoozeh SAJEDI, Pouria Pouria, Hosein DELAVAR KASMAEI

**Affiliations:** 1 Psychology and Exceptional Children Education, University of Social Welfare and Rehabilitation Sciences, Tehran, Iran,; 2 Psychologist, Pediatric Neurorehabilitation Research Center, University of Social Welfare and Rehabilitation Sciences, Tehran, Iran.; 3 Pediatric Neurorehabilitation Research Center, University of Social Welfare and Rehabilitation Sciences, Tehran, Iran.; 4 Statistics Department, University of Social Welfare and Rehabilitation Sciences, Tehran, Iran; 5 Department of Neurology, Shahid Beheshti University of Medical Sciences, Tehran, Iran.

**Keywords:** Attribution Retraining, Epileptic Children, Health

## Abstract

**Objective:**

Epilepsy is a chronic neurological disease. Evidence has indicated that epilepsy has an impact on mental and physical health of children. The present study aimed to determine the effectiveness of attribution retraining on health enhancement of epileptic children.

**Materials & Methods:**

This was an experimental study with a pre-test and a post-test design with a control group. Thirty students with epilepsy (11 female and 19 male students) were selected in convenience from Iranian Epilepsy Association. They were assigned to experimental and control groups and their mothers completed Child Health Questionnaire (CHQ-PF.28) before and after the intervention. The experimental group attended to eleven sessions (each session 45 minutes; twice a week). Subjects were trained by attribution retraining program, but control group was not. Multivariate analysis of covariance (MANCOVA) was used for analyzing the data.

**Results:**

Health (both psychosocial and physical) of experimental group enhanced significantly after the intervention sessions compared to control group.

**Conclusion:**

Attribution retraining is an effective intervention to enhance the psychosocial and physical health of epileptic children.

## Introduction

Epilepsy is one the most common chronic neurological diseases (1). It is considered as a functional disorder with permanent readiness for the onset of epileptic seizures. 

Its neurological, psychological and social consequences are determined by its characteristics. Up to 5% of a specific population will have seizures in a specific time (2). People with epilepsy are experiencing health problems, low quality of life, physical or mental, and emotional problems (3, 4), depression, anxiety (5) unpleasant physical and emotional health (6, 7), psychological and mental health problems (8) more than normal people. In many patients with epilepsy, psychosocial nature of the disease is considered as a problem (3).

Emotional and behavioral problems of children with epilepsy probably lead to physical problems (9, 10). World Health Organization defines health as a situation not only as the lack of disease or handicap but also complete physical, mental and social well-being. The physical aspect of the definition includes the lack of disability, handicap, chronic diseases, complaints and having optimal levels of energy. Its psychological aspect is consisted of an eight-divided attitude relating to five negative feelings and three positive feelings. Its social aspect includes the ability to work, satisfaction with partner, socialization and cooperation in the community (11). 

As people with epilepsy have negative self- perception, they tend to attribute their failures (which sometimes come from a neurological disease) to their inefficiency, and generalize their inefficiency and helplessness to their other experiences. They tend to loneliness and helplessness instead of solving the problem. According to the revised helplessness theory (12), individuals attribute their distress and helplessness to causes, classified according to three dimensions: internal-external, general-specific, and stable-unstable (13). The lack of control in learned helplessness situation, not only affects human health but also effects on explaining the lack of control (attribution style) (14). There are two attribution styles: optimistic and pessimistic. In the former, the negative and unpleasant events are attributed to external, unstable and specific causes. It is usually considered internal, stable and general causes for negative and unpleasant events in pessimistic attribution style (15). Cognitive and meta-cognitive methods have been used to modify the attribution style (16). One of its most effective methods is retraining attribution program. 

The purpose of attribution retraining is to help individuals to make adaptive attributions (17). The attribution style influences on psychological and physical consequences of negative life events (9). Attribution retraining can reduce physical problems (18), depression, anxiety (19) and increase performance (20). 

Considering the importance of health care for children with epilepsy and lack of similar study in Iran, the present study aimed to examine the effect of attribution retraining program on health’s components of children with epilepsy. It seems that if attribution retraining will affect children’s health in a positive way, so it can promote health and prevent physical and psychological problems. The study aimed to seek the answer for this question: whether attribution retraining can promote the health of children with epilepsy or not.

## Materials & Methods

This experimental study with a pre-test and a posttest design and a control group was conducted on 30 students with epilepsy (11 female and 19 male students) in convenience from Iranian Epilepsy Association, Iran.

They were divided to experimental (6 girls and 9 boys) and control (5 girls and 10 boys) groups. The subjects were excluded if they had physical and motor handicap, sensory inabilities (blindness, deafness), behavior disorders (conduct disorder, oppositional behavior disorder) and participation to similar intervention at the same time.

After the approval of the Ethics Committee of University of Social Welfare and Rehabilitation Sciences, Tehran, Iran, parents of children with epilepsy completed written informed consent.

All children were evaluated using the Child Health Questionnaire (CHQ-PF.28) before and after the training sessions. The questionnaire was designed by Lndygraf and Abetz (21) and measures health and of children and adolescents according to parents’ report. The questionnaire is composed of two dimensions: physical and psychosocial health. Physical health includes five components as follows: physical functioning, role functioning: physical, bodily pain, general health, and change in health. Psychosocial health includes eight components as role functioning: emotional/behavior, general behavior, mental health, self-esteem, parentalimpact: emotional, parental impact: time, time parent pressure, family activities, and family cohesion. The components, which have five options to response, are scored as 0, 25, 50, 75, and 100. The four options components are scored as 0, 33.33, 66.66, and 100. Low and high scores are considered as an indicator for weak health or well-being respectively. The questionnaire was translated to Persian and its validity was varied from 0.41 (general health) to 0.87 (physical functioning) and 0.78 for total questionnaire according to Chronbach alpha quotient. Test-retest was used to report its reliability for 3 weeks intervals. The correlation quotients varied from 0.45 (for general health) to 0.84 (for parentalimpact: time). Spearman correlation quotient was 0.65 for its content validity. Factorial analysis indicated that the questionnaire was loaded over 0.3 for almost all questions in Iranian students (21). 

Experimental group participated in 11 training sessions (twice a week; 45 min for per session) and received attribution retraining program intervention. 

Control group only received mainstream program of the Association (such as communication skills, anger management, life skills training). After the last session and six weeks later, CHQ-PF.28 was completed again by parents. 

Data were analyzed using multivariate analysis of covariance (MANCOVA). P<0.05 was considered as significant. 

Attribution retraining program was formulated according to Bandura self-efficacy theory, Seligman learned helplessness theory and Wiener theory about attribution. 

This package was arranged firstly by Golparvar ( 22) for students with dyscalculia. He reported its reliability and validity as 0.92 and 0.86, respectively. The content of each session is depicted in Table 1.

## Results

Subjects were 10 to 18 yr old (mean, 14.40, standard deviation, 2.36 for experimental group; 13.40, and 2.27 for control group, respectively). Table 2 shows descriptive indices of health dimensions of experimental and control groups in pre-test, post-test and follow-up situations.

The mean of physical and psychosocial health of experimental group have increased in post-test and follow up situations in comparison to control group (Table 2). The same findings were not found in control group.

To compare the health components of experimental and control groups according to pre-test and posttest situations, it is recommended to test whether the preassumptions of MANCOVA are guaranteed or not. By using Kolmogorov-Smirnov test, the distribution of the scores in physical and psycho-social health of epileptic children in post-test and follow-up were normal (Table 3). The results of M.Box test showed that homogeneity of variances of health components between two groups was not significant (P=0.07).

In order to determine the effect of attribution retraining program on promoting the health of epileptic children, MANCOVA was used. At first, Pillai’s trace, Wilk’s Lambda, Hotellings’ trace, and Roys’ Largest Root were significant, and indicated that overall predictive variables could differentiate between two groups.

The results of MANCOVA for comparing physical and psychosocial health of two groups are shown in Table 4. 

As reflected in Table 4, physical and psychosocial health of experimental group has increased significantly after attending to attribution retraining sessions. According to Eta quotient (H2), 75% of variations in physical and psychosocial health are due to participating in attribution retraining sessions. 

As shown in Table 5, physical and psychosocial health of experimental group has changed significantly 6 weeks follow-up attending in attribution retraining program.

## Discussion

The first finding showed that physical health of children with epilepsy increased significantly after participating in attribution retraining program sessions, which were in consistent with other studies (23, 24). These studies concluded that the attribution of the subjects changed to internal factors; therefore, they could improve their mental health. The epileptic children in present study learned to replace negative attribution with positive ones. In other words, the children learned to change their attribution from external, global, and stable factors with internal, specific, and unstable ones. Furthermore, the subjects could modify their understanding about abilities. It can be explained that epileptic children experience psychological problems due to negative selfperception.

They tend to attribute failures (which are partly because of having a neurological disease) to their incompetency, so they overestimate the failures and this leads to feel inadequacy, helplessness, and depression. 

Subsequently, they lose the opportunities to perform effectively and turn to loneliness and helplessness instead of solving the problem properly. Modification from pessimistic attribution style to optimistic one that took place in intervention sessions has led individuals to attribute unpleasant events to unstable, specific, and internal factors. Therefore, they have experienced less physical symptoms than before (13).

The second finding showed that attribution retraining has influenced on psycho- social health of children with epilepsy, which was in consistent with a previous study (25). There are three explanations for the finding: 

1) Pessimistic attribution style makes people prone to helplessness and this will be appeared as cognitive, emotional, social and physical disorders or disabilities. As a sequence, the disabilities lead to lower mental health in pessimists. 2) As pessimist people do not have confident to the consequences of their behaviors, they less try to promote their position while confronting with health problems. Therefore, this would worsen their mental health. 3) This is also plausible that the pessimistic attribution style might be resulted from illness or health problems. Since, the people divert their attention from the weaknesses and negative points to strengths and positive ones, it is probably the cause of modifying pessimistic attribution style to optimistic and this might promote psychosocial health of children with epilepsy (26, 27).

In follow-up study, attribution retraining influenced physical and psychosocial health of children with epilepsy. It can be explained that attribution retraining led to modify pessimistic attribution style to optimistic and the children could attribute their failures to external, unstable and specific factors their successes to internal, stable, and general factors respectively. Therefore, it led to promotion of their physical and psychosocial health. 

Distributing 11 attribution retraining sessions to 6 weeks (twice a week), in convenience sampling, 14 to 18 yr old children limits the generalization of the findings. It is suggested to present attribution-retraining package to parents and give responsibility to them for lengthening training course and appealing children to do more practices at home, besides demanding parents’ supervision to get more precise results. It is recommended to present the attribution-retraining program as a rehabilitative protocol for experts and clinics.

**In conclusion, **the attribution-retraining program increases self-awareness of children on their own thoughts and perceptions. As this program introduces abnormal and maladaptive behavior to individuals, they can learn how a specific attribution style forms. 

Therefore, they will know how to avoid from the behaviors and relationships, which lead to maladaptive attribution style, and as a sequence, they are able to promote their health. Attribution retraining program can be considered as a cognitive intervention by educators, psychologists and counselors who provide rehabilitation services for children with epilepsy. In addition, it probably leads to improve their quality of life, promotes their health and prevents many of their compatibility problems in adulthood.

**Table 1:** The Content of Attribution Retraining Sessions

Session	Content of each session
1	Welcoming and introducing children to each other, describing the purpose of attribution retraining
2	Informing children about their own statements about unpleasant events
3	Expressing how to analyze the events in simple speech using examples
4	Modifing attribution style of children
5	Assessing the correctness of beliefs
6	Identifing the style of self-blameless
7	Determining the role of each factor in every event
8	Training how to depute controversy attitudes
9	Explaining ABCDEF (adversity, beliefs, consequences, dispute, energizing, and feelings) in simple speech
10	Avoiding to consider events as catastrophic phenomenon
11	Acting imaginative game about the previous issues

**Table 2:** Descriptive Indices of Health Components of Experimental and Control Group in Three Situations

Group	Dimension	Pre-test		Post-test		Follow up	
		M	SD	M	SD	M	SD
Experimental	**Physical health**	26.7	7.60	29.7	7.97	28.86	6.16
	**Psycho-social health**	54.23	12.07	61.33	14.33	57.20	12.94
Control	**Physical health**	24.73	7.27	24.46	7.614	24.60	5.25
	**Psycho-social health**	49.13	8.37	48.93	7.77	45.73	6.37

**Table 3:** Kolmogorov-Smirnov test for testing normality of variables in post-test and follow-up situation

Variable	Post-test		Follow-up	
	Z	Sig	Z	Sig
Physical health	0.621	0.84	0.58	0.89
Psycho-social health	0.697	0.72	0.63	0.82

**Table 4:** Results of MANCOVA to Compare the Health Components of Experimental and Control Groups in Pre-Test and Posttest

Source of change	Variables	MS	df	F	Sig	2
Pre-test	**Physical health**	514.125	1	80.212	<0.001	0.76
	**Psycho-social health**	4.529	1	0.121	0.731	0.005
Group	**Physical health**	489.50	1	76.370	<0.001	0.75
	**Psycho-social health**	2982.8	1	79.696	<0.001	0.75

**Table 5:** Results of MANCOVA to Compare the Health Components of Experimental Group on Posttest and Follow-Up

Source of change	Variables	MS	Df	F	Sig	2
Posttest	**Physical health**	171.324	1	20.320	<0.001	0.439
	**Psycho- social health**	62.571	1	2.572	0.121	0.090
Follow-up	**Physical health**	320.773	1	38.045	<0.001	0.60
	**Psycho- social health**	2384.197	1	98..016	<0.001	0.79
